# Characterisation, prevalence and severity of skin lesions caused by ophidiomycosis in a population of wild snakes

**DOI:** 10.1038/s41598-024-55354-5

**Published:** 2024-03-02

**Authors:** Steven J. R. Allain, David I. Leech, Kevin Hopkins, Katharina Seilern-Moy, Julia Rodriguez-Ramos Fernandez, Richard A. Griffiths, Becki Lawson

**Affiliations:** 1 Durrell Institute of Ecology and Conservation, School of Anthropology and Conservation, University of Kent, Canterbury, Kent, CT2 7NR UK; 2 British Trust for Ornithology, The Nunnery, Thetford, Norfolk, IP24 2PU UK; 3 Institute of Zoology, Zoological Society of London, Regent’s Park, London, NW1 4RY UK; 4 IDEXX Laboratories Limited, Grange House, Sandbeck Way, Wetherby, West Yorkshire, LS22 7DN UK

**Keywords:** Biological techniques, Ecology, Zoology, Ecology, Environmental sciences, Diseases

## Abstract

Ophidiomycosis is an emerging infectious disease affecting wild snakes in the Northern Hemisphere. Recently confirmed in Great Britain, the prevalence, severity and significance of ophidiomycosis has yet to be characterised in free-living snakes at a population level in Europe. Therefore, a population of barred grass snakes (*Natrix helvetica*) in eastern England was monitored for three seasons (May 2019 to October 2021), to investigate the prevalence (25.5%; 191/750 snakes) and severity of skin lesions and their aetiology. The most frequently observed skin lesion characteristics were changes in scale colour, crusting, and scale margin erosion. The majority of such lesions (96.9%; 185/191 snakes) was observed on the ventral surface along the length of the body. The severity of skin lesions was considered mild in more than half of the cases (53.1%; 98/191 snakes). Predominantly, skin lesions were observed in adult snakes (72.8%; 139/191 snakes). Combined histological examinations and qPCR tests of skin lesions from *N. helvetica* sloughs and/or carcasses confirmed a diagnosis of ophidiomycosis. Further targeted surveillance, supported by molecular and histological examinations to confirm skin lesion aetiology, is required to determine the extent to which our findings reflect the occurrence of ophidiomycosis in populations within wider landscapes.

## Introduction

In recent decades, infectious diseases have emerged that threaten wildlife health, many of which are caused by fungi^1,2^. *Ophidiomyces ophidiicola* (*Oo*) is the causative agent of ophidiomycosis (commonly known as snake fungal disease or SFD)^3^. Ophidiomycosis was first detected in wild snakes in the USA in 2008^4^ and is known to affect a diversity of species occupying different habitats in North America^5^. Through retrospective examination of museum specimens, *Oo* was detected in wild snakes in the USA since at least 1945^6^. Population genetic studies of *Oo* suggest multiple recent introductions of the fungus into North America, from an as yet unsampled source population^7^. Historical detections of the disease in captive snakes on multiple continents raises the possibility that transport of snakes may have been involved in inadvertent *Oo* introductions^7–9^. The disease typically leads to skin lesion formation with variable characteristics (e.g., crusting, erosion, ulceration, granuloma formation) and severity of presentation^10^. Ophidiomycosis has the potential to impact wild snake health and lead to mortality via direct or indirect (e.g. altered behaviour leading to increased predation risk) mechanisms^3,11,12^.

Increased interest in wild reptile health led to the detection of ophidiomycosis in wild snakes in Europe^13^, and subsequently in Asia^14,15^. The disease has been diagnosed in three species of the genus *Natrix*: the barred grass snake (*Natrix helvetica*) in southern and central England^13^; the dice snake (*N. tessellata*) in the Czech Republic, Germany and Italy^13,16,17^; and the European grass snake (*N. natrix*) in Switzerland^18^. Clinical signs of the disease have also been observed in a European grass snake (*N. natrix*) from Italy^18^. Recent analysis of museum specimens found evidence of *Oo* in *N. helvetica* since at least 1959 in Italy, and 1961 in *N. tessellata* in Switzerland^19^. All three of these *Natrix* species are semi-aquatic predators, primarily of amphibians and fish^20,21^.

Phylogenetic analyses and mycological examination of *Oo* isolates derived from *N. helvetica* in England confirmed that they are genetically and phenotypically distinct from isolates in North America^7,13^. Whether *Oo* is endemic or introduced to Great Britain, or if the clade II lineages of *Oo* in North America and clade I in Europe have different rates of transmission, virulence or may result in different clinical signs remains unknown^13^.

While a growing number of studies have investigated the occurrence and impact of ophidiomycosis in free-living populations of snakes in North America^22,23^, research in Europe is relatively limited. To date, small numbers of snakes in the Czech Republic, Germany, Italy, Poland, and the Slovak Republic have tested qPCR positive for *Oo* DNA^16,24,25^. However, there is a paucity of information on disease progression and recovery in free-living snakes through serial recaptures^26^. Longitudinal population studies of individual snakes are required to develop an understanding of the epidemiology of the condition.

Here we present findings from a three-year study of a population of *N. helvetica* in eastern England in which the presence of *Oo* had been confirmed through molecular examination of lesions from multiple skin sheds collected in 2015^7,13^. Standardised survey protocols were conducted combined with opportunistic pathological examinations of snake carcasses and skin sheds to address the following: (1) to investigate the character, distribution, severity, prevalence, and aetiology of skin lesions; (2) to determine whether the presence of skin lesions is related to sex, size, slough cycle, presence of trauma, or month of capture; and (3) to compare the severity of skin lesions between serial recaptures to assess progression of, or recovery from, disease over time.

## Materials and methods

### Field surveys and sample collection

Field surveys for *N. helvetica* (hereafter grass snake) were conducted at a wetland system with a size of approximately 0.5 km^2^ in eastern England over a 3-year period, 2019–2021, during the active season (May–September). Ten grass snake carcasses and 42 snake sloughs were opportunistically collected from the site during this period and stored at − 20 °C pending pathological investigations.

A total of 69 artificial cover objects (ACOs) made of corrugated bitumen (35 of which were 2 m^2^, and 34 of which were 1 m^2^) were placed at average intervals of 52 m (range of intervals: 18.0 m to 238.6 m apart depending on topography) in potential grass snake habitat and checked one to three times a week. Morning surveys were conducted between 09.00 and 11.00 h and afternoon surveys between 16.00 and 19.00 h, when weather conditions allowed for safe working. While infrequent (< 4% of total captures), when snakes were encountered basking in the open, they were also captured and sampled using the same protocol as per snakes found under ACOs.

Snakes were captured by hand and placed in individual transparent plastic bags. During the initial capture and processing phases, snake behaviour was observed for any indicators of poor health status (e.g. lethargy, evidence of recent feeding, abnormal clinical signs). Photographs of the ventral scale patterns of captured snakes were taken to allow post-hoc individual snake identification^27^.

The entire body of each snake was inspected for evidence of skin lesions. Body length (to the nearest 0.5 cm) and body mass (to the nearest 5 g) were recorded using a tape measure and Pesola spring balance, respectively. The size class of individuals was based on their snout-to-vent length (SVL) with neonates regarded as snakes with an SVL below 20 cm, sub-adults with an SVL between 20 and 50 cm, and adults with an SVL greater than 50 cm. Snakes were sexed based on the length and profile of their tail; individuals with a noticeable vent swelling and more than 62 sub-caudal scales were identified as males^28^. The phase in the slough cycle was recorded using a three-point scale; freshly sloughed were assigned to Phase 0, dulling ventral scales to Phase 1 and opaque eyes to Phase 2.

To reduce the risk of cross-contamination and the potential exposure of clinically healthy snakes to *Oo*, clean handling procedures were used whilst processing snakes and collecting samples. Nitrile gloves were changed between handling snakes and all equipment was sanitised using dimethyl benzyl ammonium saccharinate (0.1% active ingredient; Dettol All-in-One Disinfectant spray) for a minimum contact time of 10 min^29^, rinsed with clean tap water and air dried before re-use. A 1% Virkon S (LANXESS) solution with a 10-min contact time was used to disinfect field equipment and boots at the end of each survey^29^.

If skin lesions were detected, these were swabbed in duplicate using two different MW-100 swabs (Medical Wire & Equipment), moistened with molecular-grade nuclease-free water (Thermo Fisher Scientific). Where snakes had multiple skin lesions, as many as possible were swabbed, ensuring consistency by sampling the same lesions with both swabs. Control samples were collected from every fifth snake captured without detectable skin lesions: samples were taken in duplicate from the ventral body, swabbing five times from neck to vent. Swabs were stored in a portable insulated freezer bag for the duration of the fieldwork and transferred to a dedicated − 20 °C freezer within 30 min of leaving the site.

Skin lesions were classified based on their appearance and were assigned one or more of the following seven characteristics; change of scale colour, crusting, dysecdysis, scale distortion, scale margin erosion, swelling, and ulceration. (See Supplementary Materials for definitions of skin lesion characteristics). The distribution of skin lesions was recorded for each of the five sections of the body (i.e. the head and the four quarters along the remaining body), and whether they occurred on the dorsal, ventral and/or lateral body surfaces. Photographs of the skin lesions were taken on a digital SLR camera (Pentax K-50 with an 18–55 mm lens).

A scoring system (4–16) was developed, adapted from Baker et al.^30^, to rank the severity of skin lesions based on their characteristics, number, and distribution (See Supplementary Materials). Skin lesion scores were subsequently categorised as very mild (score 4), mild (5–8), moderate (9–12), or severe (13–16). Snakes were also examined for skin injuries with an appearance consistent with trauma, e.g. punctures, lacerations, or tail tip injuries. When present, details were recorded, and photographs taken as previously described.

### Laboratory protocols

#### Pathological examinations

Post-mortem examinations (PMEs) were conducted on grass snake carcasses using a standardised protocol^13^. Briefly, systematic external and internal inspection of all body systems was conducted, supported by microbiological, parasitological, and/or histological examination as indicated based on macroscopic findings and as permitted by the state of carcass preservation. Samples of detected skin lesions were stored at − 80 °C prior to *Oo* qPCR and fixed in 10% buffered formal saline pending histological examination using routine methods.

Sloughs were measured in length (to the nearest cm) and assessed regarding their level of completeness to estimate the size class of the snake. Each slough was inspected for the presence of areas of thickening or changes of colour, potentially consistent with skin lesions. If these were present, samples were collected using a sterile scalpel blade and stored at − 20 °C pending *Oo* qPCR. If more than one lesion was present on a slough, a subset of samples was fixed in 10% buffered formal saline for histological examination.

Histopathological examination of a subset of both skin lesions from PMEs and slough lesions was conducted to screen for the presence of fungal hyphae and arthroconidia with morphology consistent with *Oo.* Haematoxylin and eosin (H&E) stain was used as routine, combined with periodic acid-Schiff (PAS) and Gram stains where indicated on the basis of case findings.

#### Real-time PCR for *Ophidiomyces ophidiicola* detection

DNA was extracted from swab samples using the protocol described by Franklinos et al.^13^; however, whilst for cotton swab tips, 60 µl of PrepMan Ultra (Applied Biosystems) was added to the sample, for skin lesion tissues only 50 µl of PrepMan Ultra was used^31^. The collected supernatant was then diluted to a 1 in 10 solution, using PCR-grade water. For negative extraction controls, sterile MW-100 swabs were used.

The *Oo* qPCR was conducted on a StepOnePlus™ Real-Time PCR Machine (ThermoFisher Scientific) as per Bohuski et al.^32^ running 60 cycles (instead of 40) and using the primers Oo-rt-ITS-F and Oo-rt-ITS-R in a 20 µl reaction with 5 µl of the 1:10 template DNA^31^. Each DNA extract was run in duplicate, and ambiguous results were repeated until consistent results were obtained: samples which generated ambiguous results after three separate runs were considered inconclusive and excluded from further analyses. Samples were considered positive if one or both of the duplicate skin swabs tested *Oo* qPCR positive with a cycle threshold (Ct) value of ≤ 36^32^.

#### Internal control PCR for host DNA detection

An internal control PCR targeting a section of the host’s Early B-cell Factor 3 (EBF3) gene was used^33^. The protocol was modified as follows: HOT FIREPol master mix was used to prepare 10 μl PCR reactions containing 0.6 μM of each primer EBF3N_F and EBF3N_R and 2 μl of either diluted template DNA (1:10) or negative controls. Thermocycling was performed using an Applied Biosystems GeneAmp 9700 PCR system and the following settings: 95 °C for 15 min, followed by 35 cycles of 95 °C for 20 s, 60 °C for 1 min, and 72 °C for 1 min, with a final 10 min at 72 °C. PCR products were visualised in a 1–2% agarose gel using 6X loading dye (GelRed, Biotium). Samples testing negative on the internal control PCR were excluded from the study.

### Statistical analysis

A total of 656 individual snakes were captured over the study period across 1,164 capture events. To maintain independence of observations within years for univariate analyses, a snake was counted just once within a year and classified as ‘without lesions’ or ‘with lesions’ based on its first capture in that year. As some snakes transitioned between ‘without lesions’ to ‘with lesions’ between years (and vice versa: see Results) recaptured snakes in subsequent years were also counted in the same way. As this means that some recaptures are included between years the total number of captures across years is higher than the number of individual snakes captured.

Binomial Generalised Linear Mixed Models (GLMMs) were used to determine which of the following fixed factors: sex, SVL, phase of the slough cycle, presence of trauma, and month of capture, were associated with the occurrence of skin lesions. As a small number of recaptured snakes were included in the analysis (that were recaptured between years), individual snake was included as a random factor within the models. The models were constructed using the data taken from the first capture of each snake caught each year from 2019 to 2021. The generalized variance-inflation factor (GVIF) was used to test for evidence of correlation between the factors to ensure there were no confounding variables (Supplementary Table S1;^34^). The Akaike Information Criterion (AIC) was used to assess the fit and rank the models^35^. Analyses were conducted using R (version 4.2.2), package lme4^36,37^.

### Ethics declaration

The protocol for snake capture and handling received ethical approval from both the School of Anthropology and Conservation (University of Kent) and the Zoological Society of London (IOZ35), and adhered to the animal ethical guidelines of both institutions. The authors complied with ARRIVE guidelines.

## Results

### Pathological examinations of carcasses and skin sloughs

Post-mortem examinations were conducted on three adult and one neonate grass snakes with skin lesions found dead over the period 2019–2021. One of the adults was female, the other carcasses were of indeterminate sex due to their size and/or state of preservation. Severe skin lesions characterised as changes of scale colour, crusting, and scale margin erosion were detected in the adult female which tested *Oo* qPCR positive (Supplementary Fig. S1): upon histopathological examination, fungal hyphae and arthroconidia with morphology consistent with *Oo* were observed, confirming a diagnosis of ophidiomycosis (Supplementary Fig. S2). Swab samples from the neonate, with evidence of mild skin lesions, and the two other adult snakes (one with mild skin lesions, the other in an advanced state of decomposition which precluded lesion scoring) tested *Oo* qPCR negative. The proximate cause of death of the adult female snake and one of the other adults remained undetermined due to a poor state of carcass preservation. The findings for the remaining two snakes with skin lesions were consistent with trauma as the cause of death.

No skin lesions were detected on the remaining six grass snakes examined post mortem, and skin swabs collected from these animals tested *Oo* qPCR negative (n = 5) or inconclusive (n = 1).

Evidence of lesions was detected in 39.0% (n = 16) of skin sloughs collected from the study site (Supplementary Fig. S3, S4), of which 56.3% (9/16 snakes) tested *Oo* qPCR positive. However, no apparent difference was noted in the appearance of skin slough lesions in *Oo* qPCR positive versus negative samples. All skin slough samples tested positive for host DNA using the internal control PCR.

Of the skin sloughs for which size data were available, lesions from a total of six sub-adult and three adult snakes tested qPCR positive for *Oo*. Samples of apparently normal skin sloughs from 10 snakes (of all size classes) screened as a control tested *Oo* qPCR negative: histological examination was conducted on samples from three of these sloughs and no evidence of microscopic lesions or fungal elements consistent with ophidiomycosis was detected.

Histopathological examination of single lesions from five sloughs revealed the presence of fungal hyphae (n = 4) and arthroconidia (n = 3) with a morphology consistent with *Oo*. Briefly, fungal hyphae had parallel walls, were occasionally septate and branching at approximately 1–4 μm in diameter, whilst the arthroconidia were rectangular in shape and approximately 2 μm × 4 μm in size, forming dense aggregates. Samples from four of the sloughs with fungal hyphae and/or arthroconidia were *Oo* qPCR positive.

### Prevalence of skin lesions

Of the 1,164 captures, 1,122 (96.4%) were of snakes found under ACOs, and 42 comprised snakes basking in the open (3.6%). Relatively more captures in the open (n = 18; 42.9%) were snakes with skin lesions compared to those under ACOs (n = 253; 22.5%; χ^2^ = 7.09, df = 1, *p* < 0.01), with the majority of captures in the open (n = 17) having occurred in 2021. The annual prevalence of skin lesions varied significantly across the study period, with 26.1% (77/295 snakes) with skin lesions encountered in 2019, 14.5% (35/241 snakes) in 2020, and 36.9% (79/214 snakes) in 2021 (χ^2^ = 29.79, df = 2, *p* < 0.01). No evidence of clinical signs indicative of general debility, such as lethargy, were observed in captured snakes with or without skin lesions.

### Character and distribution of skin lesions

**Figure 1:**
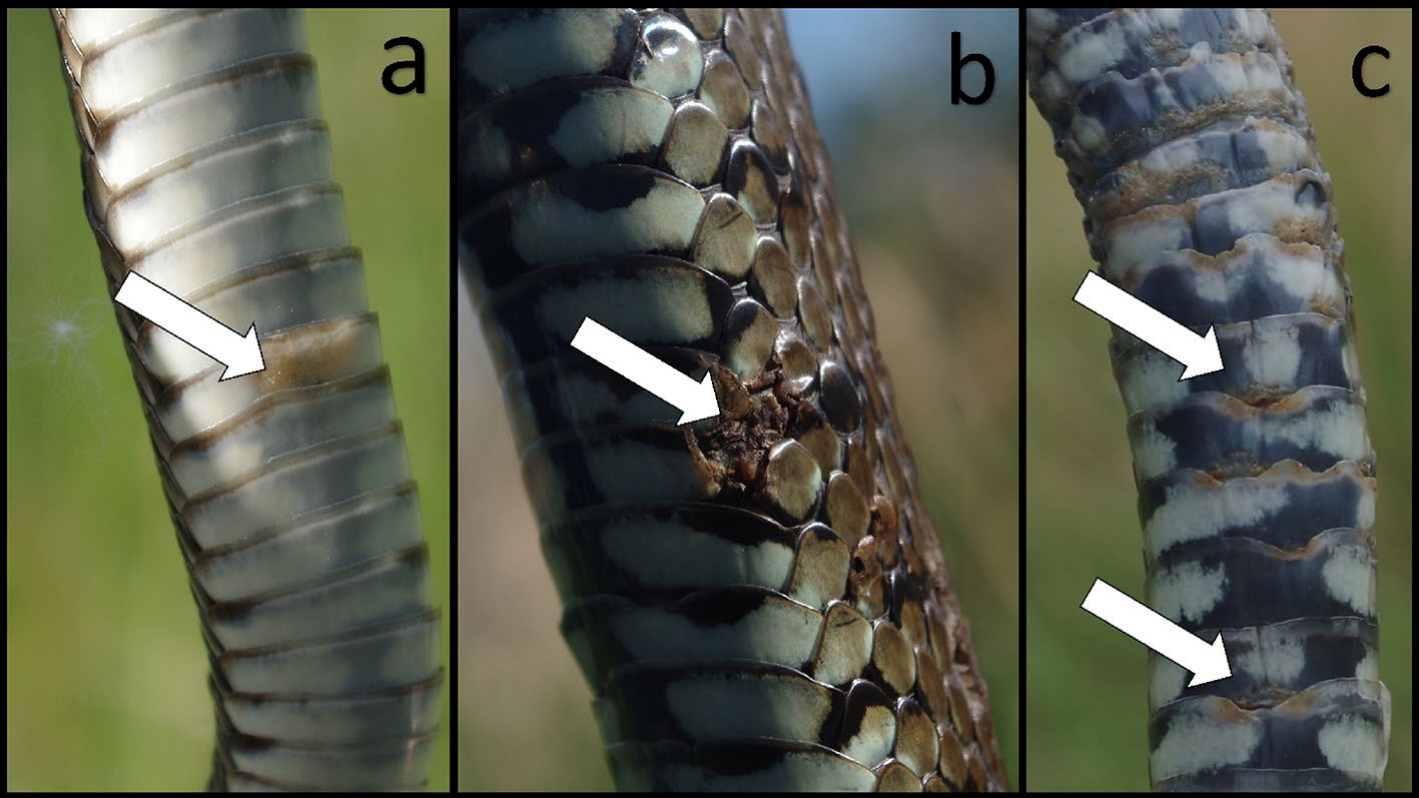
Examples of the most commonly encountered skin lesion characteristics observed in snakes during the period 2019–2021. These include a change of scale colour (**a**), change in colour with ulceration (**b**), and change in colour, crusting, and scale margin erosion (**c**). The severity score of lesions was recorded as mild (**a**), moderate (**b**), and severe (**c**).

Examples of the most frequently encountered skin lesion characteristics in grass snakes are shown in Fig. 1. The most common skin lesion characteristic observed (from the first capture of individuals with multiple recaptures) was a change in scale colour, seen in 97.9% of cases (187/191 snakes): lesions were tan, red, brown, or a combination thereof. Changes of colour were typically concurrent with crusting (89.5%; 171/191 snakes) and scale margin erosion (73.2%; 140/191 snakes). Less frequently encountered skin lesion characteristics were swelling (26.7%; 51/191 snakes) and distortion of scales (10.9%; 21/191 snakes; Supplementary Fig. S5), whilst the rarest characteristics recorded were ulceration (7.7%; 13/191 snakes) and dysecdysis (2.9%; 5/191 snakes).

**Figure 2:**
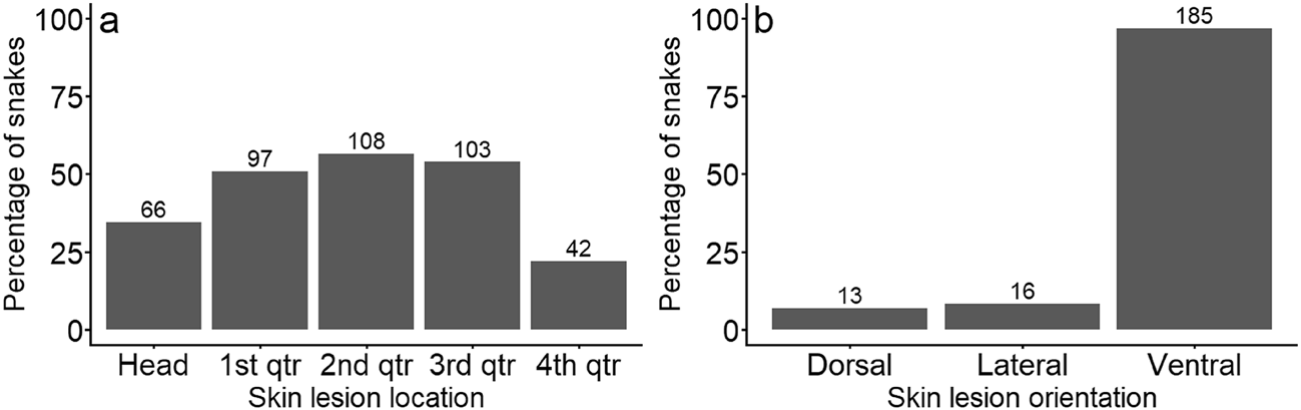
Total number and percentage of snakes with detected skin lesions categorised by location along the body length (**a**) and body surface (**b**). A total of 191 snakes with skin lesions was caught during the period 2019–2021.

The vast majority of lesions occurred on the ventral body surface with relatively even distribution along the body length (Fig. 2).

### Skin lesion severity

**Figure 3:**
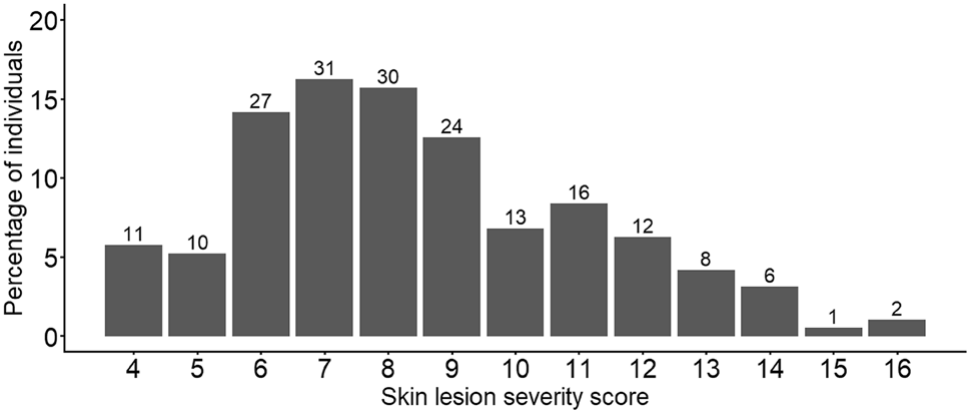
Total number and percentage of individual snakes with skin lesions with each of the severity scores during the first capture in each year (n = 191). Skin lesion severity categories were as follows: very mild (n = 11; i.e. score 4), mild (n = 98; i.e. score 5–8), moderate (n = 65; i.e. score 9–12), and severe (n = 17; i.e. 13–16). The data do not follow a normal distribution (Shapiro–Wilk: W(194) = 0.96, *p* < 0.01).

The most common category of skin lesion severity was mild (51.3%; 98/191 snakes), followed by moderate (34.0%), with very few classified as severe (8.9%) or very mild (5.8%; Fig. 3). The average severity score (including recaptures) was 8.8 in 2019, 8.2 in 2020, and 8.1 in 2021, with no significant variation by year (R^2^ = 0.623, F(1,1) = 1.69, *p* = 0.418).

### Real-time PCR results from skin swabs collected from live snakes

Over the 3-year study period, the majority of skin swabs collected from 271 live snake captures of 191 individuals with skin lesions tested *Oo* qPCR positive on the first duplicate swab (i.e. 78.7%; 215/271 snakes), and 69.3% (188/271 snakes) on the second duplicate swab, with no significant difference between the repeat swabs (paired t-test, t = 0.37, df = 4, *p* = 0.73). In contrast, a minority of skin swabs from snakes without detected skin lesions tested *Oo* qPCR positive (i.e. 2.5% (4/165 snakes) on the first duplicate and 3.0% (5/165 snakes) on the second duplicate.

The internal control PCR targeting the EBF3N gene was run on the DNA extracts from all 469 skin swabs which tested *Oo* qPCR negative. Amplification of the internal control was detected in 97.2% (226/232) of skin swabs from the first duplicate, and 100% (237/237) of swabs from the second duplicate (Supplementary Table S2). The six samples with no internal control amplification were excluded from the study.

The median Ct value of the qPCR was compared across the skin lesion severity score groupings. There was a significant difference between the Ct values for very mild, mild, moderate, and severe skin lesions (One-way ANOVA, F(3, 413) = 14.37, *p* < 0.01). Snakes with skin lesions in the greater severity score categories had lower Ct values on average than milder cases, indicating an inferred higher fungal load within the sample due to an increased quantity of *Oo* DNA (Supplementary Fig. S6).

### Seasonality of skin lesions

The detection of skin lesions in the snake population peaked in May during 2019 (44.1%, 34/77 snakes), July in 2020 (88.6%, 31/35 snakes), and June in 2021 (37.9%, 30/79 snakes). The 2020 field season was truncated due to impacts of the COVID-19 pandemic, which meant that surveys could not be conducted in May and June that year (Supplementary Table S3). There was evidence of variation between the months of capture with regards to the frequency of snakes caught with skin lesions. Across all three years, fewer snakes with skin lesions were detected after July (Tables 1, 2).

**Table 1:** A summary of the top ten Binomial Generalised Linear Mixed Models used to determine association of various factors with the occurrence of skin lesions in snakes, ranked by their Akaike Information Criterion (AIC) score.

Predictor	Number of parameters (K)	AIC	ΔAIC	Weight	Log likelihood
Month + Sex + Snout Vent Length (SVL) + Slough Cycle Phase + Trauma	12	602.92	0.00	0.79	− 289.25
Month + Sex + SVL + Slough Cycle Phase	11	605.51	2.59	0.21	− 291.58
SVL + Slough Cycle Phase + Trauma	7	620.40	17.59	0.00	− 303.13
Slough Cycle Phase + SVL	6	621.11	18.19	0.00	− 304.50
Month + SVL	7	643.80	40.89	0.00	− 314.83
Sex + SVL	4	656.76	53.85	0.00	− 324.36
SVL + Trauma	4	657.59	54.67	0.00	− 324.77
SVL	3	658.86	55.94	0.00	− 326.41
Month + Slough Cycle Phase	9	727.66	124.74	0.00	− 354.71
Month + Trauma	7	736.83	133.91	0.00	− 361.34

**Table 2:** Parameter estimates, standard errors, z-values, and P-values for the most supported generalised linear mixed model (Month + Sex + Snout Vent Length + Slough Cycle + Trauma), investigating which factors are most associated with skin lesions. ‘May’ is used as the baseline for comparing variation between months; ‘Female’ is used as the baseline for Sex; ‘Absent’ is used as the baseline for Trauma. Significant results are marked with an asterisk.

Parameter	Estimate	Standard error	z-value	*p* value
(Intercept)	− 3.83	0.64	− 6.03	< 0.01
June	0.53	0.35	1.52	0.13
July	− 0.79	0.32	− 2.49	0.01*
August	− 0.62	0.39	− 1.59	0.11
September	− 0.88	0.49	− 1.76	0.07
Sex: Male	0.46	0.22	2.09	0.04*
Snout Vent Length	0.07	0.01	9.22	< 0.01*
Slough Cycle: Phase 0	− 1.40	0.52	− 2.72	< 0.01*
Slough Cycle: Phase 1	− 0.11	0.54	− 0.21	0.83
Slough Cycle: Phase 2	− 0.01	0.56	− 0.02	0.98
Trauma: Present	0.81	0.37	2.18	0.03*

### Size and sex of snakes with skin lesions

**Figure 4:**
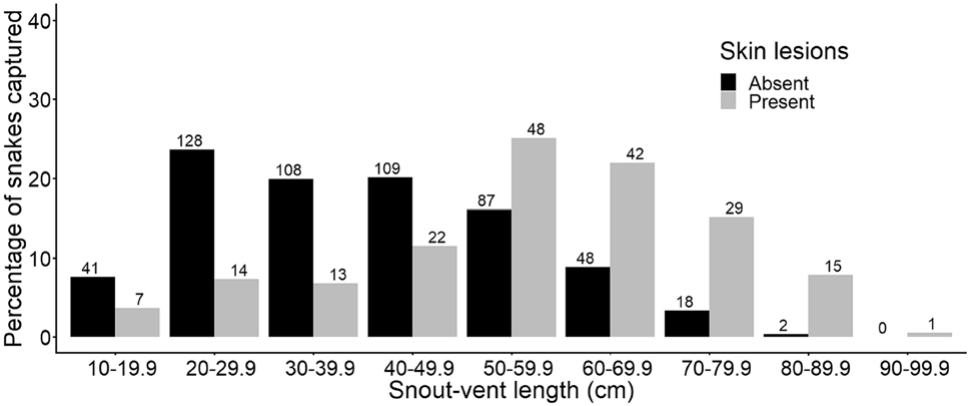
Total number and percentages of size classes of snakes captured 2019–2021 inclusive, comparing snakes with skin lesions (n = 191) to snakes without skin lesions (n = 541). There were 18 individuals that were captured but escaped during measuring, so no measurements were taken.

The majority of snakes captured without skin lesions had a SVL that is consistent with sub-adults. However, most of the snakes with skin lesions had a SVL consistent with adults (72.8%, 139/191; Fig. 4). Consequently, there was a significant association between the presence of skin lesions and size of the snakes (Tables 1, 2).

The number of male and female snakes without skin lesions was almost identical, with 272 females (50.3.%) and 269 males (49.7.0%) captured during the sampling period, whereas there was a slightly higher number of male snakes seen with skin lesions (54.9%; 105/191 snakes) (Tables 1, 2). There were a small number of individuals (n = 18) where the sex could not be determined.

### Slough cycle

**Figure 5:**
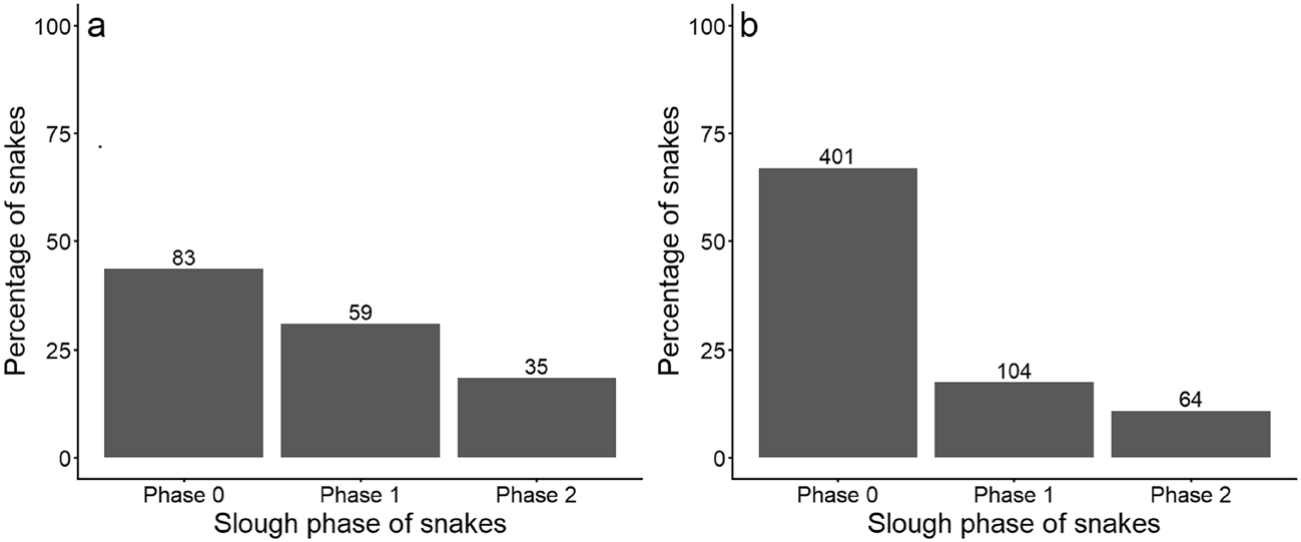
Total number and percentages of snakes, captured 2019–2021 inclusive, in each phase of the slough cycle, comparing snakes with skin lesions (**a**) to snakes without skin lesions (**b**). The total numbers of snakes displayed above do not match those reported earlier as these figures are based on the first encounter of each individual each season, which does not take into account any transition in disease state.

There was a significant association between the slough cycle and the presence of skin lesions (Tables 1, 2). Although most snakes were captured during the long phase between sloughing (i.e. phase 0), relatively more snakes with lesions than without lesions were captured in phases 1 and 2 (Fig. 5). A total of 24 individuals from both groups of snakes were removed from the analysis, as they were captured during the pilot stage of the project, before individuals were assigned to the three phases of the slough cycle.

### Trauma

Skin injuries consistent with trauma were observed in 13.6% (26/191 snakes) with concurrent skin lesions over the study period, of which the most commonly recorded were damage to the tail (n = 19), lacerations (n = 3), puncture wounds (n = 3), and combinations of the above (n = 1) (Supplementary Fig. S7). An additional 20 snakes (3.7%, 20/541) without skin lesions had injuries consistent with trauma. The type of these traumatic injuries was similar to those seen in snakes with skin lesions; damage to the tail (n = 15), lacerations (n = 2), puncture wounds (n = 2), and combinations of the above (n = 1). Signs of trauma were detected more frequently in snakes with skin lesions than those without (Tables 1, 2).

### Modelling predictors of skin lesions

Investigation of the generalised GVIF indicated that there were no significant correlations between the covariates as demonstrated by GVIF scores between 1 and 2 (Supplementary Table 3). The model that included ‘individual’ as a random factor was a better fit than the best-fitting model with fixed effects only, indicating that accounting for individual variation was important (χ^2^ = 211.9, df = 11, *p* < 0.01).

The best-fitting GLMM model was that which contained all potential predictor variables: month, sex, SVL, phase of slough cycle, and trauma (Table 1). Indeed, all of these variables were significant predictors of the presence of skin lesions (Table S1). Models that excluded one or more of these variables were ranked lower than the top model by AIC, and were therefore a poorer fit (Table 1). In summary, the prevalence of skin lesions was lower in July compared to the baseline month of May; males had a slightly higher prevalence of lesions than females; the presence of lesions increased with snake SVL; fewer snakes displayed skin lesions in Phase 0 of the slough cycle than in the other phases; and skin lesions occurred slightly more often in snakes with evidence of trauma than those without.

### Comparison of severity score between recaptures

**Figure 6:**
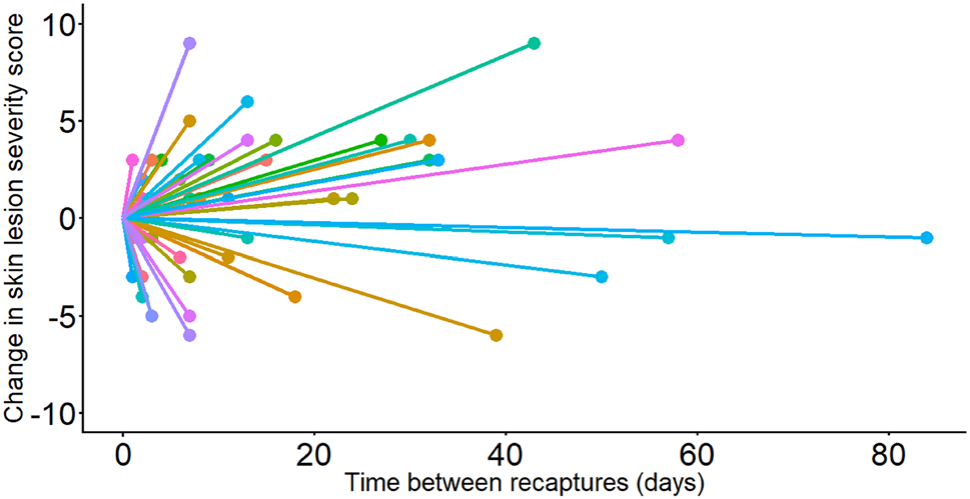
A graphical representation of the change in skin lesion severity score between the initial capture and a subsequent recapture of 36 snakes within the same year (n = 54). Each colour represents a different individual snake. Eighteen snakes were caught on at least three occasions: change in skin lesion score between within-year paired captures are represented in two (or more) lines of the same colour.

A total of 54 within-year recaptures of 36 individuals (with 18 snakes being caught on at least three occasions) was recorded over the three-year study: the average intervals between recaptures varied per year, with 14.5 days (3–32; n = 15) in 2019, 6.3 days (2–17; n = 7) in 2020, and 17.4 days (1–84; n = 32) in 2021. An increase in score severity between paired capture intervals was recorded in 33 of these cases, representing potential disease progression, while a decrease was observed in 21 cases, consistent with recovery (Fig. 6). The severity of skin lesions in 25 individuals remained relatively constant (i.e. within 1–3 points) over a period of 1 to 84 days, whereas in 11 individuals a greater difference in score (i.e. ≥ 4 point difference) was observed over a period that varied from 2 to 58 days, within the year of capture. There was a small number (n = 12) of snakes with apparent recurrence of skin lesions between years (i.e. snake had skin lesions on initial capture, was subsequently observed with no detectable skin lesions in the same year, and then recaptured with skin lesions in a subsequent year).

## Discussion

During this three-year study, we found a prevalence of skin lesions in a population of wild grass snakes of 25.5% (191/750 snakes). The most common skin lesion characteristics were changes in colour, crusting, and scale margin erosion. Whilst skin lesion appearance for ophidiomycosis is not pathognomonic, the common characteristics of changes in scale colour, crusting and scale margin erosion observed in captured live animals, were similar to those described in the same species with confirmed ophidiomycosis at PME^13^. Our results represent the first field data for this species, which are similar to findings in other *Natrix* spp.^16,24,25^, but contrast markedly with published observations in some other wild snake species (e.g. cottonmouths (*Agkistrodon piscivorus*), corn snakes (*Pantherophis guttatus*), and timber rattlesnakes (*Crotalus horridus*)) in North America, where facial deformities and swelling are observed with ophidiomycosis^3,38,39^.

The high percentage of skin lesions testing *Oo* qPCR positive in this study is comparable with findings in other snake species (such as common watersnake (*Nerodia sipedon*) and queen snake (*Regina septemvittata*)) with similar ecological niches in aquatic habitats from North America^40^. Dillon et al.^23^ noted that exposure to *Oo* is likely linked to habitat type, with marsh habitats having the highest probability of disease manifestation in the form of skin lesions in the eastern fox snake (*Pantherophis vulpinus*) in Ontario, Canada, based on qPCR detection. The semi-aquatic nature of *N. helvetica* (and other closely related *Natrix* spp. in Europe) may therefore predispose this species to ophidiomycosis, in contrast to snakes from drier habitats, such as the European adder (*Vipera berus*), in which ophidiomycosis has yet to be diagnosed in Great Britain.

The majority of skin lesions observed in snakes in this study occurred on the ventral surface, which has greatest contact with the ground and may therefore be more exposed to the fungal spores of *Oo* within the environment. A similar ventral distribution of skin lesions has previously been reported by Chandler et al.^41^ in the eastern indigo snake (*Drymarchon couperi*) in Georgia, USA. Scarification of the stratum corneum has been noted to facilitate infection with *Oo* in challenge studies^3^, with mechanical abrasion caused by a snake navigating its environment likely to have a similar result.

In the majority of cases with observed skin lesions with an appearance consistent with ophidiomycosis, the presence of *Oo* was confirmed by qPCR, supporting the hypothesis that ophidiomycosis is their primary cause (Supplementary Table S2). Additionally, the observed significant association between the higher severity score of skin lesions and lower Ct value, which indicates more *Oo* spores to be present, further supports ophidiomycosis as the aetiology. Following the terminology developed by Baker et al.^30^, cases of snakes with qPCR positive skin lesions can be categorised as apparent ophidiomycosis. However, histopathological investigation of skin lesions in living snakes to further investigate causality was not considered feasible or ethical, due to the requirement of biopsies and/or scale clips. Therefore, confirmation of presence of *Oo* DNA as well as fungal elements invading the skin tissue could only be attempted in six cases (one carcass and five skin sloughs): one carcass and three sloughs had confirmed ophidiomycosis, one slough had possible/apparent ophidiomycosis, and one slough had *Oo* present (according to classification developed by Baker et al.^30^). The small number of qPCR positive swabs from *N. helvetica* without detected skin lesions may indicate subclinical infection or environmental contamination and are classified as *Ophidiomyces* present^30^.

Where qPCR failed to detect *Oo* DNA despite the presence of skin lesions (i.e. 20.4% of cases), potential false negative qPCR results have to be considered. It is also possible that other potential pathogens, such as *Paranannizziopsis* spp. were involved, which have the ability to cause skin disease in snakes^42,43^. Disease surveillance with a combination of ancillary diagnostic tests to investigate the aetiology of such lesions should continue to gain a better insight into the range of aetiologies for skin disease in wild European snakes.

None of the snakes with detected skin lesions, even those classified as severe, exhibited altered patterns of behaviour consistent with systemic ill health, or a moribund state. This contrasts with reports of affected snakes in North America (e.g. *C. horridus* and *Agkistrodon piscivorus*) where clinical signs of ill health, such as emaciation, lethargy, or a lack of a righting reflex, were observed^10,39^. Collectively, our findings indicate that direct mortality caused by ophidiomycosis in grass snakes may be low, however, further research is required to explore this hypothesis. Radiotelemetry to track affected individual snakes could provide a reliable method to further monitor disease progression and survival.

The increased occurrence of traumatic injuries in snakes with skin lesions observed in this study suggests a higher frequency of encounters with predators, therefore supporting the theory of increased basking behaviour in snakes with ophidiomycosis and subsequent higher risk of predation^12^. It has been suggested that such behaviour might relate to higher frequencies of thermoregulatory postures^44^, or to the increased metabolism needed to promote sloughing required for clearing fungal infections^3^. While puncture wounds are highly suggestive of predation, the cause of tail damage and lacerations, which were classed as predation injuries in this study, is less certain, although may still be linked to predation^45,46^.

We further found that skin lesions were more likely to be associated with the later phases of the slough cycle, potentially because affected snakes may slough more frequently, which may then inadvertently lead to higher predation rates due to snakes being unable to see for comparatively longer periods of time or having to slough in riskier microhabitats^47^. Alternatively, the association of skin lesions with the later stages of sloughing could simply be due to the more delicate condition of the skin at this stage.

In contrast to Lind et al.^44^, who found no link between sex or reproductive status and *Oo* detection in pygmy rattlesnakes (*Sistrurus miliarius*), we observed that male grass snakes had a higher likelihood of presenting with skin lesions compared to females. Since male snakes are more active, ranging over larger distances while searching for suitable females during the breeding season^48^, they may have an increased likelihood of ventral scale scarification and exposure to *Oo* in the environment. Adult snakes were more commonly found to have skin lesions compared to juveniles or subadults. When investigating the presence of *Oo* in North American water snakes (*Nerodia* spp*.*), Harding et al.^49^ also found that adults had the highest prevalence of *Oo* qPCR positive skin lesions.

Our data indicate a strong seasonality of ophidiomycosis in grass snakes with approximately 30% of detections of skin lesions coinciding with their peak active season in May and June. This is a similar outcome to other studies where skin lesions were found to be more prevalent in snakes following emergence from hibernation^10,23^. For the two study years with complete field season data available, skin lesions peaked in May as snakes were encountered post-hibernation, with affected individuals less frequently encountered later in the year. This may be linked to an increase in ambient temperature in the summer months, which may aid in enhancing immune function and clearing infection, or it may be more closely correlated to hibernation and/or the seasonality of the slough cycle of snakes^50–52^.

Through the use of serial recaptures, we found evidence of different rates of skin lesion progression as well as apparent recovery from ophidiomycosis among individual snakes. In cases where potential disease recurrence was observed, fungal hyphae may have remained in the skin (either by penetrating deeper layers or through the presence of improperly sloughed skin), leading to subsequent reappearance of skin lesions^3^.

 The extent to which the findings of this study reflect the occurrence of ophidiomycosis affecting *N. helvetica* populations (or other *Natrix* spp.) throughout Europe requires further investigation. Future targeted surveillance programmes with active sampling strategies for *Oo* detection in wild snakes might optimise the efficiency and maximise the likelihood of pathogen detection by adopting a risk-based approach and focusing on sampling of adult snakes early in the season soon after emergence from hibernation. In conclusion, there is a need for further surveillance to improve assessment of the impact of disease on free-living snake populations.

### Supplementary Information

Supplementary Information.

## Data Availability

The datasets used and/or analysed during the current study are available from the corresponding author on reasonable request.
