# Supplementary material for: Characterisation, prevalence and severity of skin lesions caused by ophidiomycosis in a population of wild snakes
Source: Sci Rep. 2024 Mar 2;14:5162. doi: 10.1038/s41598-024-55354-5 (PMC10908839; doi:10.1038/s41598-024-55354-5)
Supplement: Supplementary file 1 — Supplementary Information. [file 41598_2024_55354_MOESM1_ESM.docx]

**Characterisation, prevalence and severity of skin lesions caused by ophidiomycosis in a population of wild snakes**

**Supplementary Materials**

**Skin lesion categorisation and severity scoring scheme in barred grass snakes (*Natrix helvetica*)**

Skin lesions were categorised on the basis of the following features:

1. Location
2. Number
3. Size
4. Characteristic

- Change of colour: where scale(s) is/are a different colour to adjacent scales of the same type (brown/red/tan).
- Crusting/scab: where scale(s) has/have become thickened and/or hardened.
- Distorted scales: scale(s) that appears misaligned with surrounding scales and/or are misshapen. Evidence of scarring may be apparent.
- Dysecdysis: Abnormal sloughing with evidence of skin retention.
- Scale margin erosion: Irregular prolife of scale margin(s) due to tissue loss/erosion.
- Swelling: Swelling of scale(s), raised in profile with visible doming.
- Ulceration: The complete loss of scale(s) or dermis that expose the underlying tissues.

Categories were not mutually exclusive since multiple skin lesions may be present.

The following scoring system was developed to grade skin lesion severity based on these categories, adapted from Baker *et al*., (2019). Points were assigned based on highest the possible value in each category:

- Location: dorsal, lateral or ventral surface of body (one of the options) = 1 point; dorsal, lateral or ventral surface of body (multiple options) = 2 points; head (but not around mouth or eyes) = 3 points; head (around mouth or eyes) or cloaca = 4 points
- Number: single lesion = 1 point; 2–4 lesions = 2 points; 5–9 lesions = 3 points; 10 or more lesions = 4 points
- Size (total size (diameter of round lesions, length of linear lesions) of all lesions present): < 10 mm = 1 point; 10–50 mm = 2 points; 50-100 mm = 3 points; >100 mm = 4 points (The size of each skin lesion was either estimated in the field, or later measured post-hoc using ImageJ and photographs of the skin lesions, taken alongside a ruler).
- Characteristic: change of colour/swelling = 1 point; crusting/distorted scales/scale margin erosion (<5 mm) = 2 points; scale margin erosion (>5 mm) = 3 points; dysecdysis/ulceration = 4 points


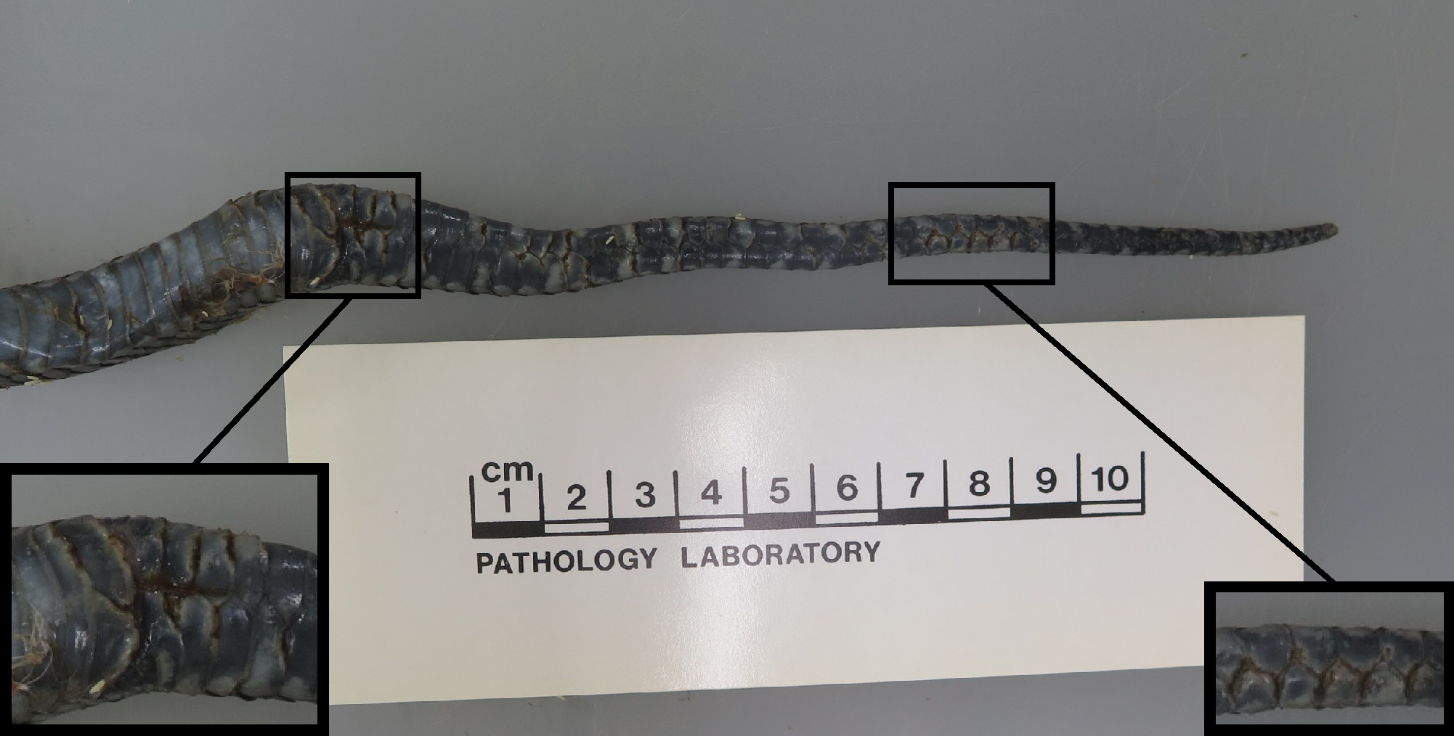


**Figure S1:** Skin lesions along the tail section of the adult female barred grass snake (*Natrix helvetica*) that tested *Oo* qPCR positive, with additional histopathological confirmation of ophidiomycosis (see Fig. S2). Severe skin lesions characterised as change of colour, crusting and scale margin erosion are present; skin lesions around the cloaca and further along the tail are shown as inset, at higher magnification.

**
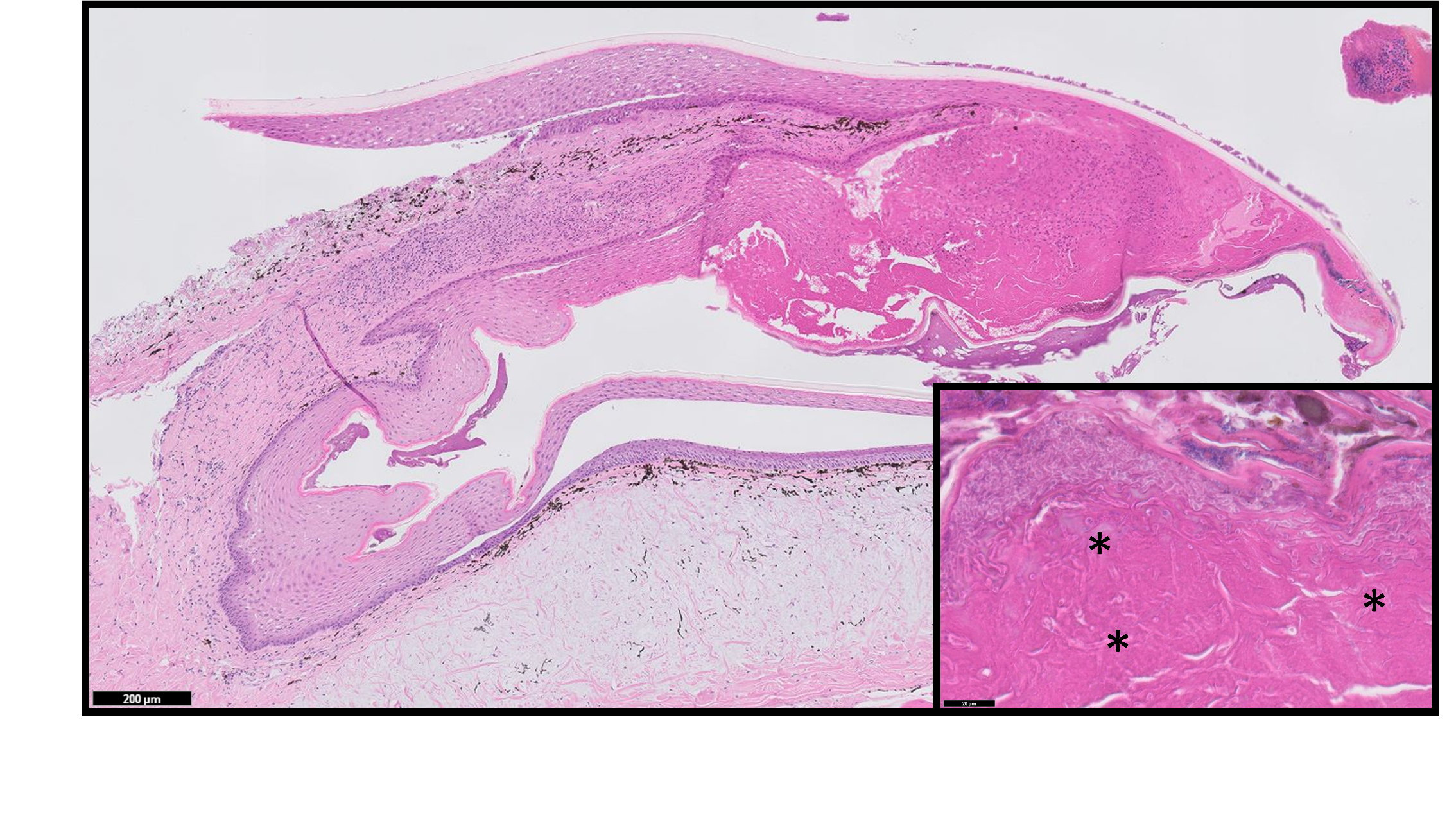
**

**Figure S2:** Photomicrograph of skin lesions from an adult female barred grass snake (*Natrix helvetica*). There is an area of epidermal thickening and necrosis, with abundant superficial fungal hyphal elements (asterisks), and associated dermatitis. H&E stain, 5x magnification and 20x for inset.

**
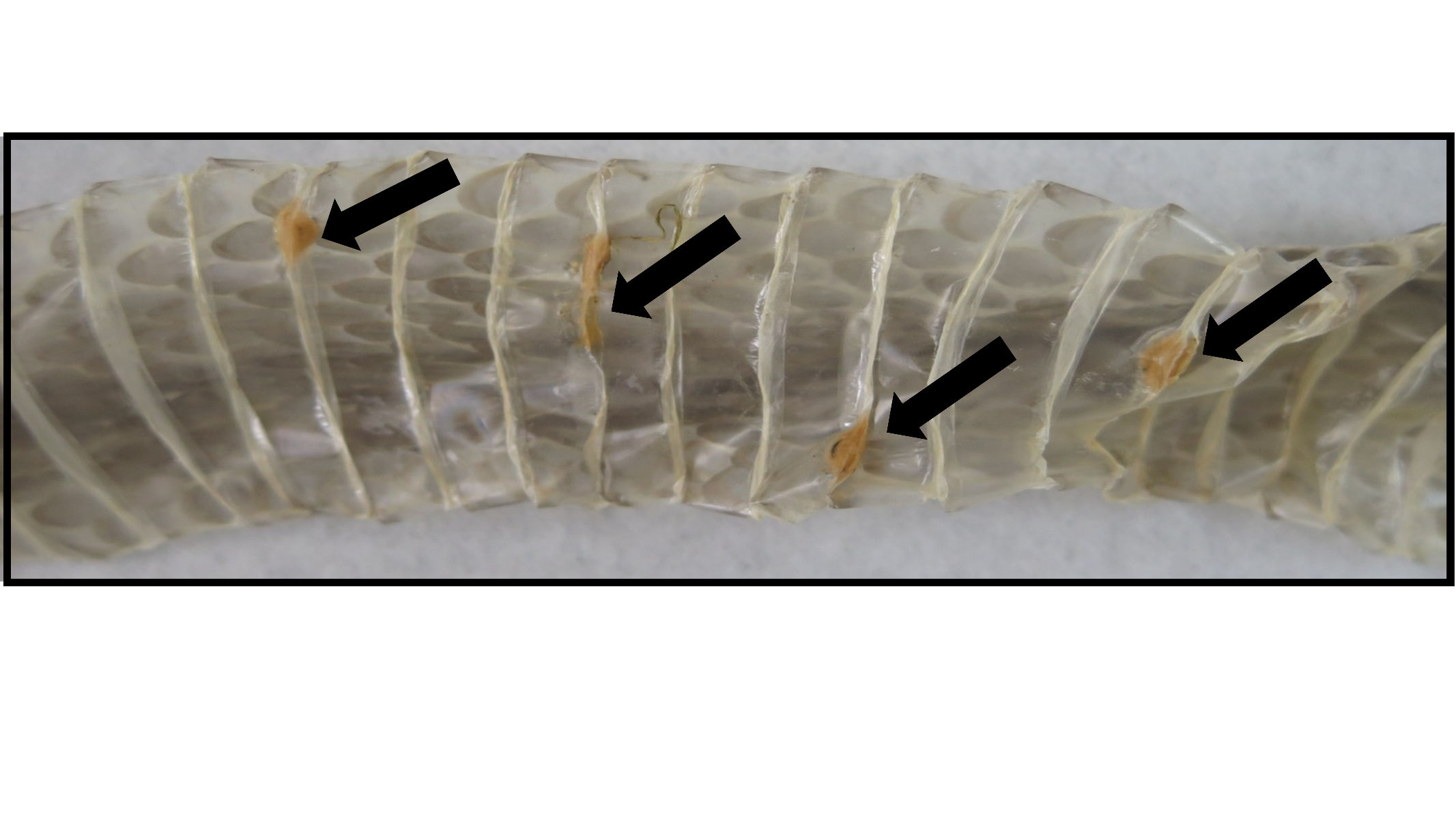
Figure S3:** A slough from a barred grass snake (*Natrix helvetica*) with multiple tan-coloured lesions (indicated by arrows) which tested *Oo* qPCR positive.

**
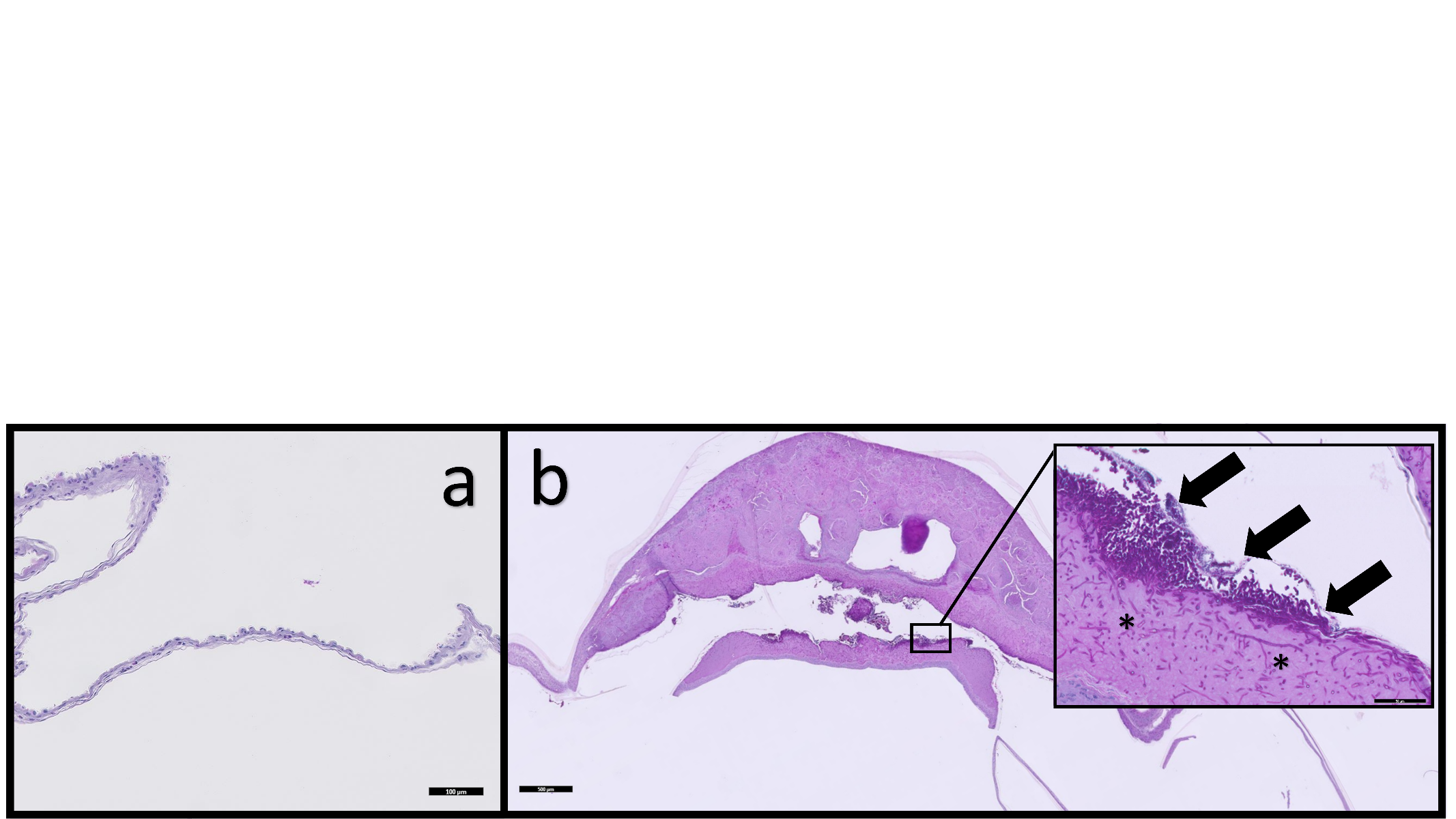
**

**Figure S4:** Photomicrograph of barred grass snake (*Natrix helvetica*) skin shed. Normal skin shed (a), and areas of thickening with abundant fungal elements, hyphae (asterisks) and arthroconidia (arrows) (b). PAS stain, 10x magnification (a), 2x magnification (b), and 40x magnification for inset.


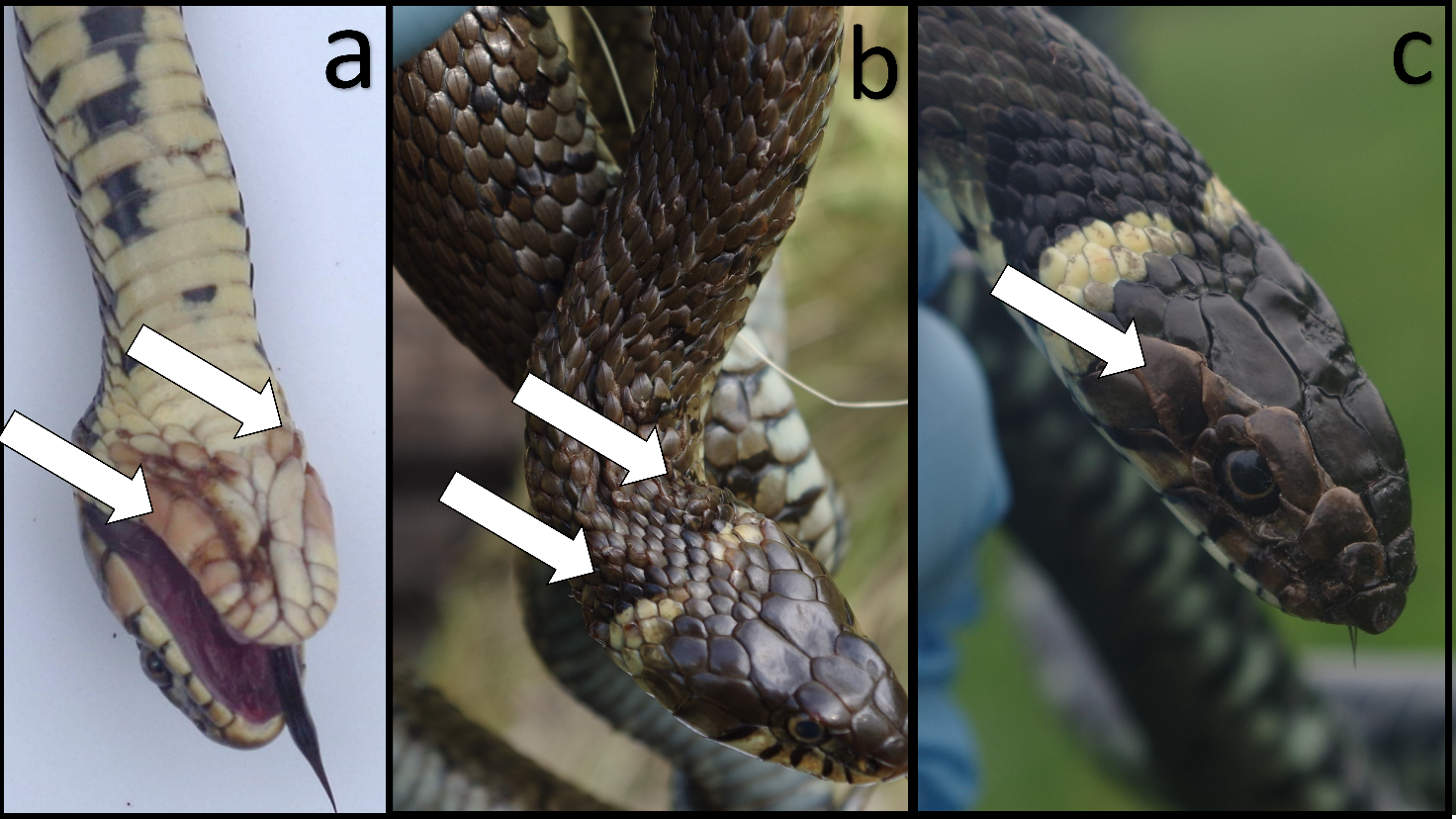


**Figure S5:** Examples of the more rarely encountered skin lesion characteristics observed in barred grass snakes (*Natrix helvetica*). These include swelling (a), distorted scales (b), and dysecdysis (c).


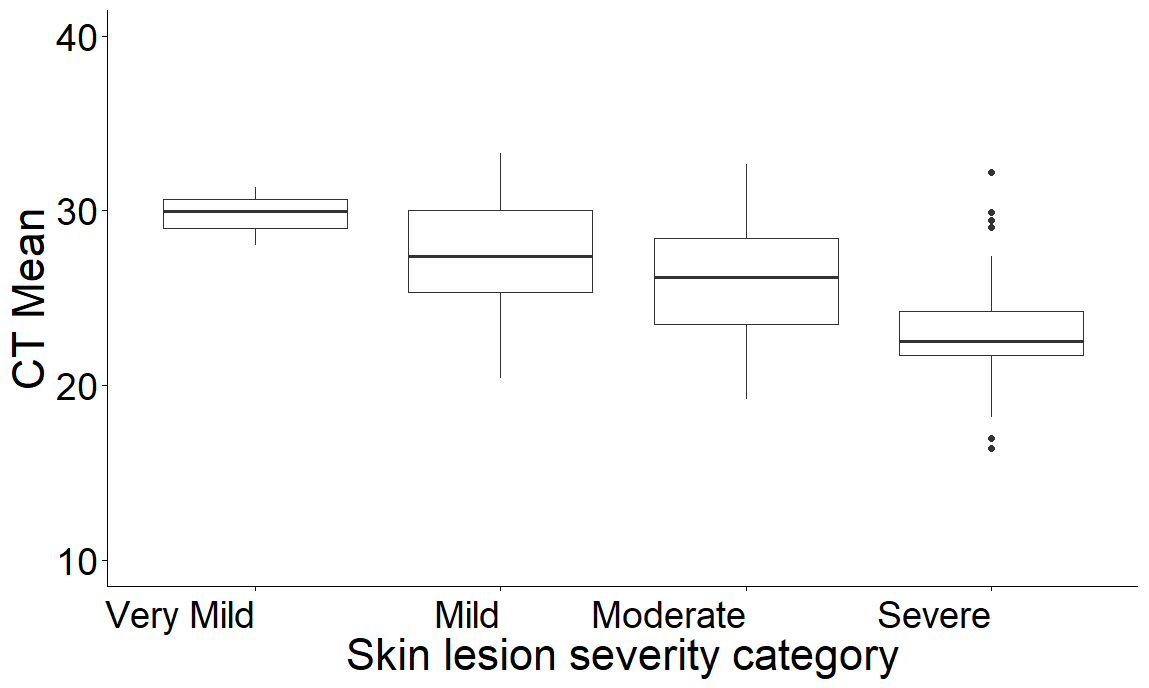


**Figure S6:** Cycle threshold (Ct) values from each qPCR positive sample from both swab duplicates, within the four skin lesion severity categories. Results from 414 qPCR tests are included, corresponding to snakes in each of the four skin lesion severity categories listed. Plotted are the means, interquartile ranges, and total ranges, and outliers (black dots).


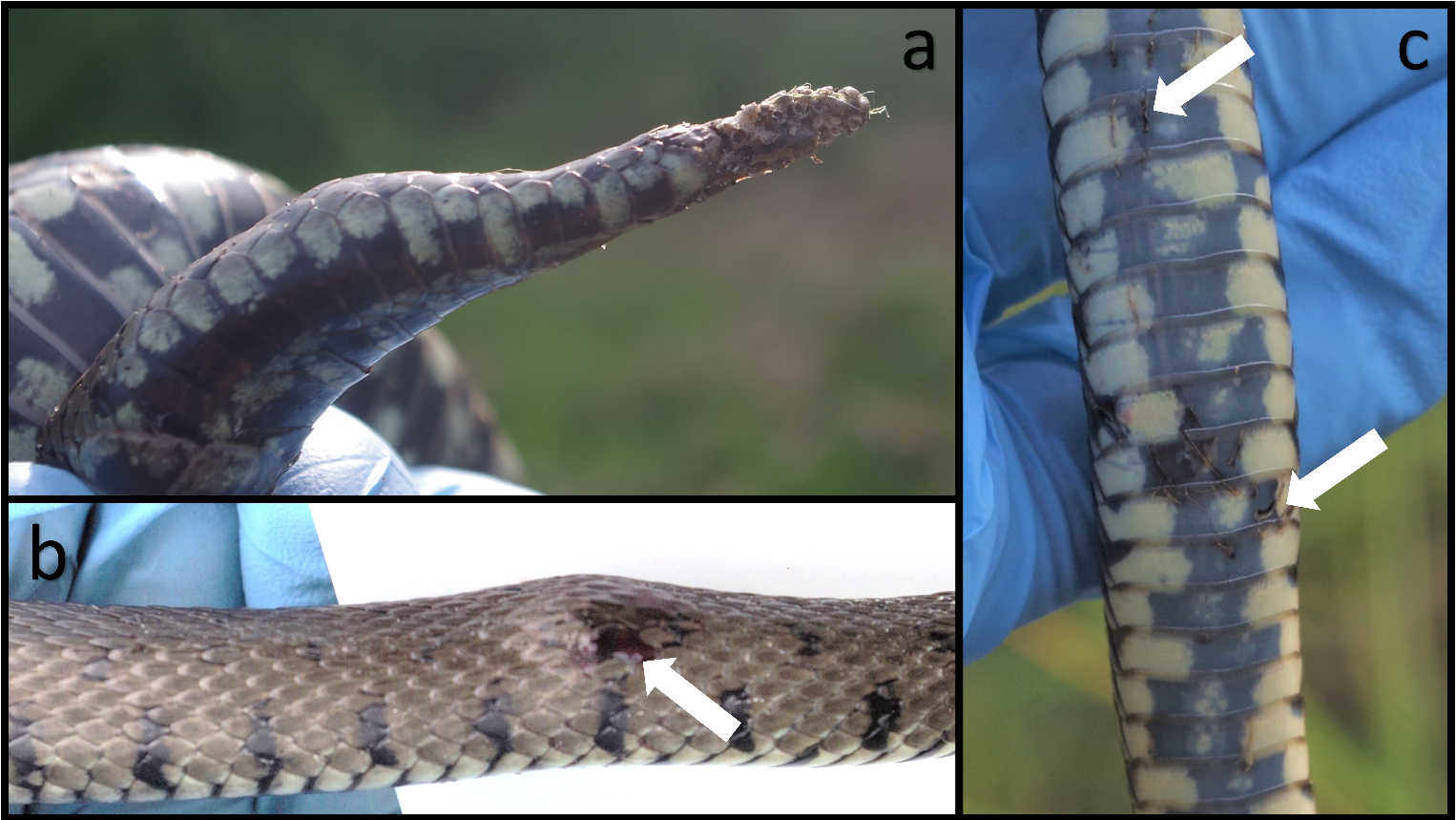


**Figure S7:** Examples of skin injury consistent with trauma observed in the barred grass snake (*Natrix helvetica*), the end of the tail missing with localised necrosis (a), puncture wound with bleeding and localised swelling consistent with inflammation (b), and linear lacerations

to the underside of a snake (c).

**Table S1:** The generalised variation inflation factor (GVIF) for each of the covariates within the most supported model described in Table 1.

| **Factor** | **GVIF** | **Degrees of Freedom** |
| --- | --- | --- |
| Month | 1.48 | 4 |
| Sex | 1.07 | 1 |
| SVL | 1.19 | 1 |
| Slough cycle | 1.48 | 3 |
| Trauma | 1.07 | 1 |

**Table S2:** Results of qPCR for detection of *Ophidiomyces ophidiicola* DNA in skin swabs from barred grass snakes (*Natrix helvetica*), 2019-2021 inclusive. For the purpose of this study, barred grass snakes were considered positive if one or both of the duplicate skin swabs tested *Oo* qPCR positive.

| **Year** | **With skin lesions** | | **Without skin lesions** | |
| --- | --- | --- | --- | --- |
| **2019** | Positive | 86 (86.0%) | Positive | 2 (3.3%) |
|  | Negative | 14 (14.0%) | Negative | 59 (96.7%) |
| **2020** | Positive | 41 (82.0%) | Positive | 3 (5.1%) |
|  | Negative | 9 (18.0%) | Negative | 56 (94.1%) |
| **2021** | Positive | 105 (86.8%) | Positive | 3 (6.3%) |
|  | Negative | 16 (13.2%) | Negative | 45 (93.7%) |

**Table S3:** The frequency of captures of the barred grass snake (*Natrix helvetica*) with skin lesions, during each month of the active field season across the three years that data were collected, compared to the number of captures each month. The percentage of total captures of snakes with skin lesions each month is provided in brackets.

|  | **2019** | **2020** | **2021** |
| --- | --- | --- | --- |
| **May** | 34/101 (33.7%) | NA | 9/29 (31.0%) |
| **June** | 23/110 (20.9%) | NA | 30/102 (29.4%) |
| **July** | 13/101 (12.9%) | 31/262 (11.8%) | 15/131 (11.5%) |
| **August** | 5/92 (5.4%) | 3/62 (4.8%) | 17/71 (23.9%) |
| **September** | 2/33 (6.1%) | 1/33 (3.0%) | 8/37 (21.6%) |
